# Spatial metabolomics to profile metabolic reprogramming of liver in *Schistosoma japonicum*-infected mice

**DOI:** 10.1017/S0031182025000162

**Published:** 2025-09

**Authors:** Yu Zhang, Ming Luo, Junhui Li, Chen Guo, Jie Jiang, Ying Zhang, Gao Tan, Xiaoli Liu, Yingzi Ming

**Affiliations:** 1 Transplantation Center, The Third Xiangya Hospital, Central South University, Changsha, HN, China; 2 NHC key laboratory of translational research on transplantation medicine, The Third Xiangya Hospital, Central South University, Changsha, Hunan, China; 3 Clinical Research Center for Infectious Diseases in Hunan Province, The Third Xiangya Hospital, Central South University, Changsha, Hunan, China; 4 Zhongnan Hospital of Wuhan University, Institute of Hepatobiliary Diseases of Wuhan University, Transplant Center of Wuhan University, Wuhan, China; 5 Department of Infectious Diseases, Hospital of the Hunan Provincial Corps of the People’s Armed Police Force, Changsha, Hunan, China

**Keywords:** mass spectrometry imaging, schistosomiasis, spatial metabolomics

## Abstract

Schistosomiasis is a parasitic disease that imposes a significant burden on society. The eggs are the primary pathogenic factor in schistosomiasis, and their accumulation in liver could lead to the formation of granulomas and liver fibrosis. However, the metabolic changes in liver resulting from schistosomiasis remain poorly understood. We established a mouse model of schistosomiasis japonica, where the eggs accumulate in the liver and form egg granulomas. We used mass spectrometry imaging to analyze the differences in metabolites among various liver regions, including the liver tissue from normal mice, the liver area outside the granulomas in schistosomiasis mice, and the granuloma region in schistosomiasis mice. There were significant differences in metabolites between different liver regions, which enriched in metabolic pathways such as the biosynthesis of unsaturated fatty acids, taurine and hypotaurine metabolism, glycerophospholipid metabolism, glycolysis/gluconeogenesis, purine metabolism, arachidonic acid metabolism, and bile secretion. In normal liver tissue, higher concentrations of oleic acid (FA (18:1)), eicosapentaenoic acid (FA (20:5)), and L-glutamine were observed. In liver regions outside the granulomas, D-glucose and pyruvic acid were elevated compared to those in normal mice. Taurine increased in the liver of schistosomiasis. Meanwhile, there were elevated uric acid and spermidine in the egg granulomas. We employed mass spectrometry imaging technology to investigate metabolic reprogramming in liver of *Schistosoma japonicum*-infected mice. We explored the spatial distribution of differential metabolites in liver of schistosomiasis including unsaturated fatty acids, taurine, glutamine, spermidine, and uric acid. Our research provides valuable insights for further elucidating metabolic reprogramming in schistosomiasis.

## Graphical abstract

**Figure:**
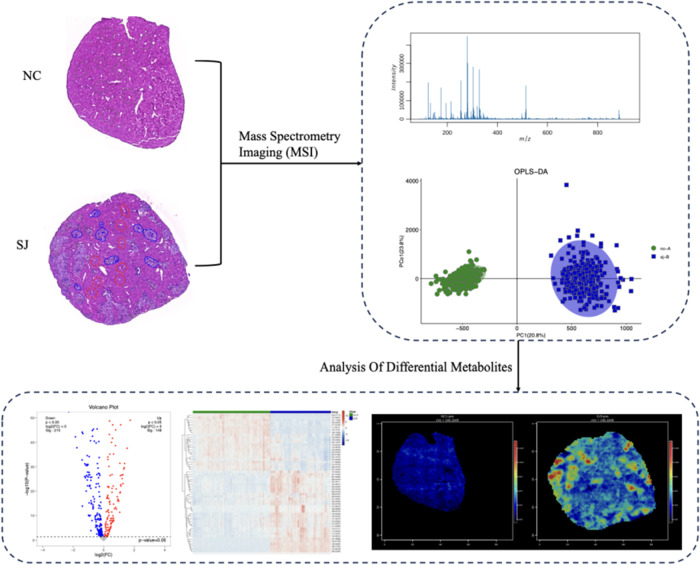

## Introduction

Schistosomiasis is a neglected tropical disease that imposes a significant burden on society, affecting over 200 million people worldwide (McManus et al., 2018). The schistosomes that infect humans include three main species: *Schistosoma haematobium* (*S. haematobium), S. mansoni*, and *S. japonicum* (Wang et al., 2013). Acute schistosomiasis occurs when humans contact with water containing cercariae. Inside the host, cercariae develop into adult worms, which then produce eggs deposited in tissues (Ross et al., 2007). Praziquantel is an effective treatment for schistosomiasis, but it primarily kills adult worms and is less effective against eggs. Eggs are a major pathogenic factor in chronic schistosomiasis (Colley et al., 2014). *S. haematobium* mainly affects the urinary system, while *S. mansoni* and *S. japonicum* primarily affect the mesenteric venous system (Zhang et al., 2007). Eggs deposited in the liver attract various immune cells, leading to the formation of egg granulomas and promoting liver fibrosis (Chuah et al., 2014). Schistosoma-associated liver fibrosis could cause portal hypertension, resulting in splenomegaly, ascites, and esophageal and gastric varices, which are significant factors contributing to the poor prognosis of patients with schistosomiasis (Li et al., 2019).

The liver is a crucial organ for metabolism, acting as a major hub for energy synthesis and conversion. Liver maintains blood sugar levels by regulating the synthesis and breakdown of glucose (Han et al., 2016). Additionally, the liver plays a vital role in lipid metabolism, overseeing the synthesis and degradation of fatty acids as well as the production and transport of cholesterol (Badmus et al., 2022). It synthesizes essential plasma proteins, including albumin, clotting factors, and transport proteins, and liver is also involved in amino acid metabolism by converting ammonia into urea for excretion through the urea cycle (Paulusma et al., 2022). Schistosomiasiscould affect the glucose and lipid metabolism (Cai et al., 2021). Clinical results have showed that the serum levels of total cholesterol (TC), triglycerides (TG), and HDL-C in the infected individuals with schistosomiasis were lower than those in the uninfected group, which indicate a lower risk of developing atherosclerosis (Shen et al., 2015; Zinsou et al., 2020). Additionally, host insulin sensitivity increases in schistosomiasis, with upregulation of glycolysis-related gene expression and downregulation of gluconeogenesis gene expression (Xu et al., 2019). The deposition of eggs in the liver causes granuloma formation and liver fibrosis, and the impact of these changes on liver metabolism requires further investigation.

Metabolites are small molecules generated during metabolic processes within organisms. These include carbohydrates, lipids, amino acids, and nucleotides, all of which are essential for cellular physiological and biochemical functions (Husted et al., 2017). Liquid chromatography-mass spectrometry (LC-MS) has become the predominant analytical technique in metabolomics research, offering insights into thousands of metabolites in a single analysis. It is particularly valuable for metabolomic analyses of biological samples such as blood, urine, and cells (Chen et al., 2023). However, LC-MS methods have limitations when analyzing metabolites in complex and heterogeneous tissues or organs. During the tissue homogenization, metabolite extraction, purification, and enrichment, it results in the loss of spatial distribution information of metabolites within the tissues. Mass spectrometry imaging (MSI) is an advanced molecular imaging technique that directly provides detailed structural, quantitative, and spatial distribution information of metabolites (Unsihuay et al., 2021). In this study, we employed MSI to explore the metabolic reprogramming of liver in *S. japonicum*-infected mice.

## Methods

### Mice model of S. japonica

Male C57BL/6 mice, 6 weeks old, were purchased from Hunan Slack Jingda Experimental Animal Company. At the Experimental Animal Center of Central South University, the mice were housed under specific pathogen-free conditions and free access to food and water. The protocols for the animal experiments were approved by the local ethics committee for The Third Xiangya Hospital of Central South University (Changsha, China). *S. japonicum*-infected Oncomelania hupensis snails were supplied by Hunan Provincial Institute of Parasitic Diseases in China. Following the induction of cercarial release, the mice were infected percutaneously with fresh cercariae (25 ± 2). On day 35 post-infection, the mice were administered praziquantel (500 mg/kg, once daily for 2 days). The mice were sacrificed until week 8 after infection.

### Sample preparation

Fresh liver tissue from *S. japonicum*-infected mouse (*n* = 1) and normal mouse (*n* = 1) was embedded and then sectioned into approximately 10 μm thick slices using a cryostat microtome (Leica CM 1950, Leica Microsystem, Germany). The sections were thaw-mounted on positive charge desorption plate (Thermo Scientific, U.S.A.) and stored at − 80°C until needed. Prior to MSI analysis, the slices were desiccated at − 20°C for 1 hour and then at room temperature for 2 hours before being analyzed. Additionally, a neighboring section was reserved for hematoxylin–eosin (H&E) staining.

### Data acquisition and MSI analysis

MSI analysis was conducted following the methods described by Luo et al. (2013). The experiment was performed using an AFADESI-MSI platform (Beijing Victor Technology Co., LTD, Beijing, China) in tandem with a Q-Orbitrap mass spectrometer (Q Exactive, Thermo Scientific, U.S.A.). For negative ion mode, the spray solvent was acetonitrile (ACN)/H2O (8:2), and for positive ion mode, the spray solvent was ACN/H2O (8:2, 0.1% FA). The solvent flow rate was set at 5 μL/min, the transfer gas flow rate at 45 L/min, and the spray voltage at 7 kV. The distances between the sample surface and the sprayer, as well as between the sprayer and the ion transfer tube, were maintained at 3 mm. The MS resolution was set to 70,000, with a mass range of 70–1000 Da. The automated gain control target was set to 2E6, with a maximum injection time of 200 ms. The S-lens voltage was set to 55 V, and the capillary temperature to 350°C. During MSI scanning, the speed in the X direction was 0.2 mm/s, and the vertical step size in the Y direction was 100 μm.

### Data processing

The raw mass spectrometry files in .raw format were converted to .imzML format using imzMLConverter and then imported into MSiReader (an open-source interface to view and analyze high resolving power MS imaging files on Matlab platform; Race et al., 2012). Ion images were reconstructed following background subtraction using the Cardinal software package (Bemis et al., 2015). All MS images were normalized using total ion count normalization for each pixel (Wang et al., 2022). To precisely extract region-specific MS profiles, high spatial resolution H&E images were used for matching. Differences in metabolites across various tissue microregions were initially screened using supervised statistical analysis methods, specifically orthogonal partial least squares discrimination analysis (OPLS-DA). The variable importance of projection (VIP) values derived from the OPLS-DA model were used to describe the overall contribution of each metabolite to group differentiation. Metabolites with VIP values greater than 1 were considered potential differentiating metabolites. Subsequently, a two-tailed Student’s *t*-test was employed to verify whether the differences in metabolites between groups were significant. Finally, metabolites with VIP values greater than 1.0 and *P* values less than 0.05 were selected as differentiating metabolites.

### Analyte identification

The ions detected by AFADESI were annotated by the pySM pipeline and an in-house SmetDB database (Lumingbio, Shanghai, China; Palmer et al., 2017).

## Results

### Sptial multivariate analysis of liver in S. japonicum-infected mice

**Figure 1.**
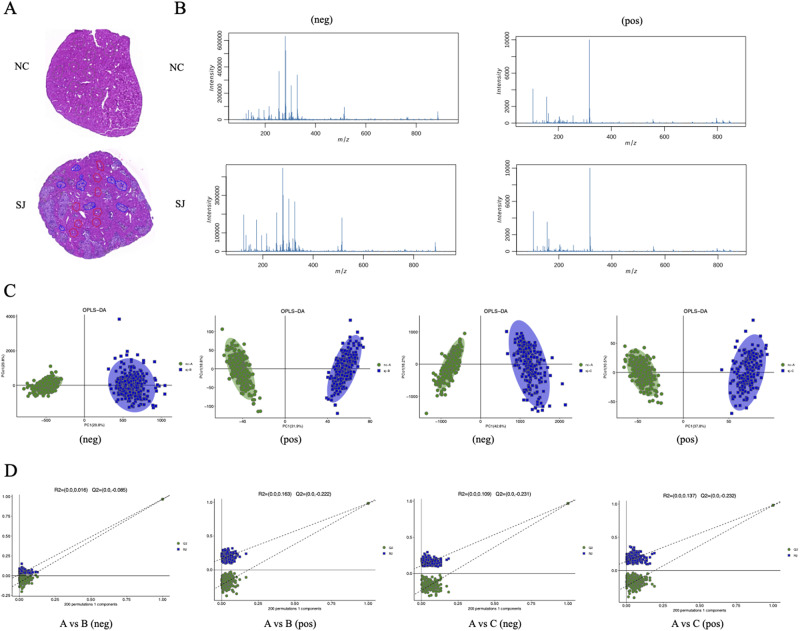
Spatial multivariate analysis of liver in *S. japonicum*-infected mice. (A) HE staining of the liver of normal mouse and mouse with schistosomiasis. (B) Mass spectrometry of the liver of normal mouse and mouse with schistosomiasis under negative and positive ion modes. (C) OPLS-DA model analysis between Areas A and B, as well as between Areas A and C, under negative and positive ion modes. (D) Assessment of OPLS-DA model using 200 rounds of response permutation testing (RPT). Abbreviations: NC, normal control mouse; SJ, *S. Japonicum*-infected mice; neg, negative ion modes; pos, positive ion modes; OPLS-DA, orthogonal partial least squares discriminant analysis; A, the liver tissue from normal mice (Area A); B, the liver area outside the granulomas in mice of schistosomiasis (Area B); C, the granuloma region in mice of schistosomiasis (Area C).

We constructed *S. japonicum*-induced mice, and the eggs deposited in livers were surrounded by a large number of immune cells, forming egg granulomas (Figure 1A). The liver tissue can be categorized into the following regions: (A) the liver tissue from normal mice, (B) the liver area outside the granulomas in mice of schistosomiasis, (C) the granuloma region in mice of schistosomiasis. To investigate the metabolic changes of liver in schistosomiasis, we conducted MSI analysis on the livers of both normal and Schistosoma-infected mice. The mass spectrometry spectra revealed the intensity of the mass-to-charge ratios (m/z) for livers of *Schistosoma*-infected mice and normal mice negative and positive ion modes (Figure 1B). These spectra were generated by averaging the m/z intensity across each pixel. We employed multivariate statistical analysis to compare the different regions. In both negative and positive ion modes, there were significant differences in metabolic distribution between the normal liver area (Area A) and the liver area outside the granulomas (Area B), as well as between the normal liver area (Area A) and the granuloma region (Area C; Figure 1C). We evaluated the quality of the model using 200 rounds of response permutation testing. The Q2 values for all group comparisons were less than zero, indicating that the OPLS-DA model was not overfitted (Figure 1D).

### Differential metabolites in the liver of schistosomiasis

**Figure 2.**
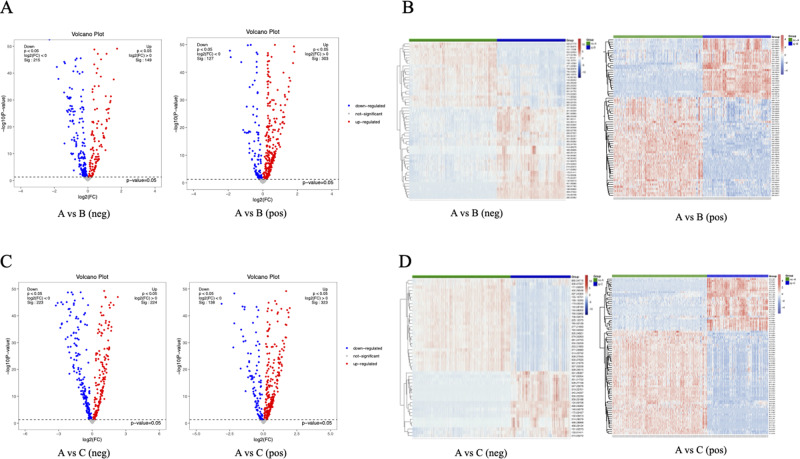
Differential metabolites in the liver of schistosomiasis. (A) Volcano plots of differential metabolites between area a and area b under negative and positive ion modes. (B) Heatmaps of differential metabolites between area a and area b with VIP values > 1 and *t*-test *P* values < 0.05 under negative and positive ion modes. (C) Volcano plots of differential metabolites between area a and area c under negative and positive ion modes. (D) Heatmaps of differential metabolites between Areas A and C with VIP values > 1 and *t*-test *P* values < 0.05 under negative and positive ion modes. Abbreviations: neg, negative ion modes; pos, positive ion modes; A, the liver tissue from normal mice (Area A); B, the liver area outside the granulomas in mice of schistosomiasis (Area B); C, the granuloma region in mice of schistosomiasis (Area C).

We identified differential metabolites across different regions. Compared to Area B, Area A exhibited 215 downregulated and 149 upregulated metabolites in the negative ion mode, and 127 downregulated and 303 upregulated metabolites in the positive ion mode (Figure 2A). In the OPLS-DA analysis, we used VIP values to assess the influence and explanatory power of each metabolite’s expression pattern on sample classification. To identify biologically significant differential metabolites, we combined multivariate and univariate analyses. We selected metabolites with VIP values greater than 1 in the PLS-DA model and a *P* value less than 0.05 in the *t*-test. Based on this, the analysis in the negative ion mode revealed 34 downregulated and 22 upregulated differential metabolites, while in the positive ion mode, there were 46 upregulated and 27 downregulated differential metabolites (Figure 2B). When comparing Area A with Area C, we found 223 downregulated and 224 upregulated differential metabolites in the negative ion mode, and 323 upregulated and 138 downregulated in the positive ion mode (Figure 2C). Among these metabolites with VIP values > 1 included 20 downregulated and 28 upregulated in the negative ion mode, and 55 upregulated and 28 downregulated in the positive ion mode (Figure 2D).

### Functional enrichment analysis of differential metabolites

**Figure 3.**
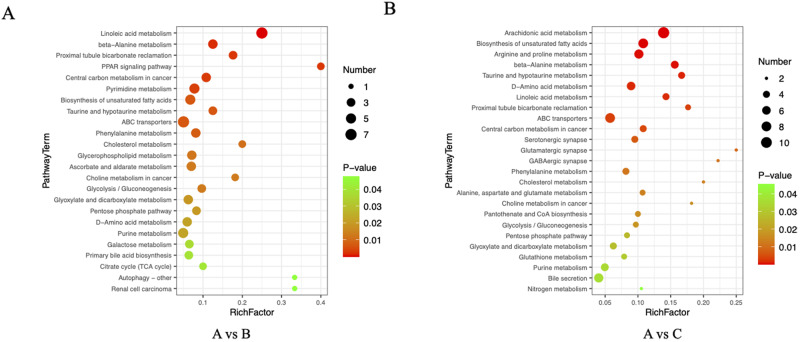
Functional enrichment analysis of differential metabolites. (A) Bubble plot of KEGG functional enrichment for differential metabolites with VIP values > 1 and *t*-test *P* values < 0.05 between Area A and Area B. (B) Bubble plot of KEGG functional enrichment for differential metabolites with VIP values > 1 and *t*-test *P* values < 0.05 between Areas A and C. Abbreviations: A, the liver tissue from normal mice (Area A); B, the liver area outside the granulomas in mice of schistosomiasis (Area B); C, the granuloma region in mice of schistosomiasis (Area C).

We integrated differential metabolites with VIP values > 1 and *P* values < 0.05 from the *t*-test, including those identified in both the negative and positive ion modes. To further understand the functions of these differential metabolites, we performed KEGG functional enrichment analysis. Compared to Area A, the functional enrichment of differential metabolites in Area B was associated with linoleic acid metabolism, biosynthesis of unsaturated fatty acids, taurine and hypotaurine metabolism, glycerophospholipid metabolism, glycolysis/gluconeogenesis, purine metabolism, and primary bile acid biosynthesis (Figure 3A). When comparing Area A with Area C, the functional enrichment of differential metabolites was linked to arachidonic acid metabolism, biosynthesis of unsaturated fatty acids, arginine and proline metabolism, taurine and hypotaurine metabolism, and bile secretion (Figure 3B).

### *In situ* distribution of crucial differential metabolites in the liver of schistosomiasis

**Figure 4.**
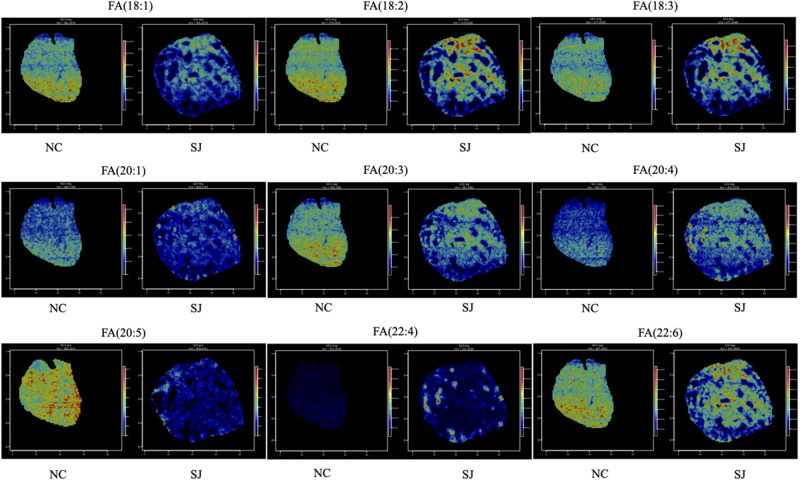
*In situ* distribution of unsaturated fatty acids in the liver of schistosomiasis. The spatial distribution of differential metabolites including unsaturated fatty acids FA (18:1), FA (18:2), FA (18:3), FA (20:1), FA (20:3), FA (20:4), FA (20:5), FA (22:4), and FA (22:6) in the livers of normal mice and mice with schistosomiasis. Abbreviations: NC, normal control mouse; SJ, *S. Japonicum*-infected mice; neg, negative ion modes; pos, positive ion modes; FA, fatty acid.

**Figure 5.**
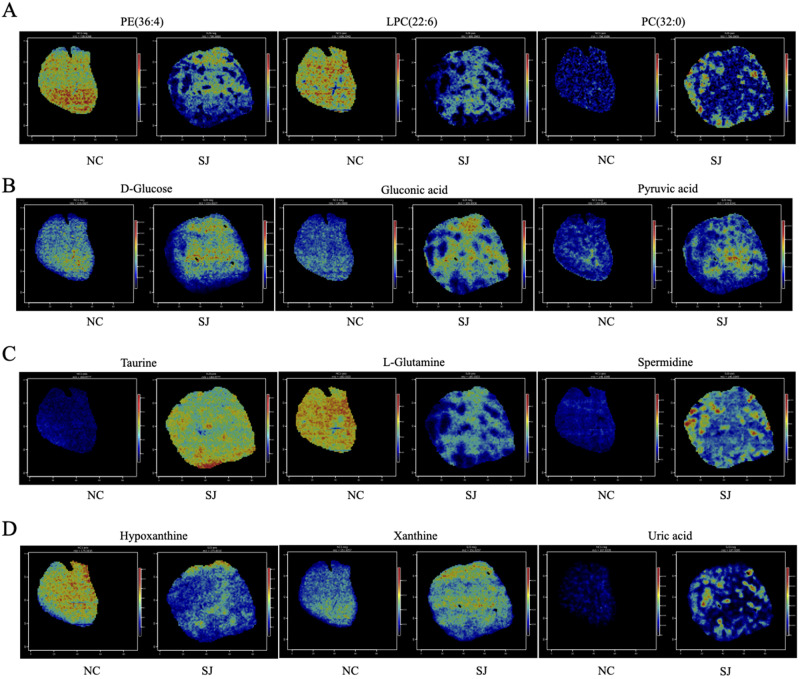
*In situ* distribution of crucial differential metabolites in the liver of schistosomiasis. (A) The spatial distribution of PE (36:4), LPC (22:6), and PC (32:0) in the livers of normal mice and mice with schistosomiasis. (B) The spatial distribution of D-glucose, gluconic acid, and pyruvic acid in the livers of normal mice and mice with schistosomiasis. (C) The spatial distribution of taurine, L-glutamine, and spermidine in the livers of normal mice and mice with schistosomiasis. (D) The spatial distribution of hypoxanthine, xanthine, and uric acid in the livers of normal mice and mice with schistosomiasis. Abbreviations: NC, normal control mouse; SJ, *S. Japonicum*-infected mice; neg, negative ion modes; pos, positive ion modes; PE, phosphatidylethanolamine; LPC, lysophosphatidylcholine; PC, phosphatidylcholine.

Unsaturated fatty acids, including FA (18:1), FA (18:2), FA (18:3), FA (20:1), FA (20:3), FA (20:4), FA (20:5), FA (22:4), and FA (22:6), exhibited significant differences across various liver regions. In normal liver (Area A), FA (18:1) and FA (20:5) were abundant. In the liver area outside the granulomas (Area B), FA (18:2) and FA (18:3) were predominant. In the granuloma region (Area C), the primary unsaturated fatty acids were FA (20:4) and FA (22:4) (Figure 4). Phospholipids have multiple functions, including maintaining the normal structure and function of biological membranes, participating in cell signaling, and facilitating the absorption and transport of fats and fat-soluble substances. In normal liver tissue (Area A), PE (36:4) and LPC (22:6) were present in high levels, while PC (32:0) was predominantly found in the granuloma region (Area C; Figure 5A). In the liver area outside the granulomas (Area B), the levels of D-glucose, gluconic acid, and pyruvic acid were elevated (Figure 5B). Taurine were notably higher in the liver of schistosomiasis (Areas B and C), while L-glutamine was increased in normal liver (Area A). Spermidine levels was abundant in the granuloma region (Area C; Figure 5C). Additionally, there were differences in purine metabolism. Hypoxanthine was higher in normal liver (Area A), whereas xanthine was elevated in the liver of schistosomiasis (Areas B and C), and uric acid was increased around the granuloma region (Area C; Figure 5D).

## Discussion

Schistosomiasis is a parasitic disease that triggers immune responses and inflammatory changes, while also impacting the metabolism of host. Traditional LC-MS techniques are widely employed for metabolite analysis; however, they analyze whole tissues and lack spatial resolution. Schistosome eggs deposited in the liver lead to the formation of granulomas and liver fibrosis. In schistosomiasis, there are notable metabolic differences between granuloma regions and the surrounding liver tissue. To better detect and understand the progression of schistosomiasis, we employed MSI to investigate the metabolism in the liver of schistosomiasis.

We conducted a comparative analysis of the liver in normal mice (Area A), the liver areas outside granulomas in schistosomiasis (Area B), and the granuloma regions in schistosomiasis (Area C). Significant differences were observed in the metabolites among these regions. The pathways enriched with differential metabolites were primarily associated with the biosynthesis of unsaturated fatty acids, taurine and hypotaurine metabolism, glycerophospholipid metabolism, glycolysis/gluconeogenesis, purine metabolism, primary bile acid biosynthesis, arachidonic acid metabolism, and bile secretion. Studies showed that genes involved in metabolic processes within hepatic granulomas, compared to uninfected controls, were associated with the metabolism of xenobiotics, sterols, fatty acids, and vitamin (Chuah et al., 2013). In our research, there were significant differences in the abundance of unsaturated fatty acids among the three regions. In normal liver tissue (Area A), oleic acid (FA (18:1)) and eicosapentaenoic acid (FA (20:5)) were present in higher concentrations. Some research show that oleic acid has anti-apoptotic effects and helps alleviate endoplasmic reticulum stress, playing a crucial protective role in liver cells (Liu et al., 2023). The levels of oleic acid were reduced in liver of schistosomiasis, indicating that liver cells might be more vulnerable to damage in schistosomiasis. Current research also shows that eicosapentaenoic acid could inhibit lipogenesis and promote liver fatty acid oxidation (Sugiyama et al., 2008). In the liver of schistosomiasis, linoleic acid (FA (18:2)) and α-linolenic acid (FA (18:3)) were more highly expressed in the liver regions outside the granulomas (Area B). Linoleic acid and α-linolenic acid are classified as polyunsaturated omega-6 and omega-3 fatty acids, respectively. Linoleic acid and its oxidized metabolites might exacerbate liver damage by promoting inflammatory responses, and the accumulation of linoleic acid in liver of schistosomiasis could be a potential cause of liver damage (Warner et al., 2017). In contrast, α-linolenic acid has different effects; it could improve insulin resistance and reduce inflammatory responses (Gonçalves et al., 2018). In the granuloma regions, the unsaturated fatty acids identified included arachidonic acid (FA (20:4)) and (FA (22:4)), both of which are polyunsaturated omega-6 fatty acids. The deposition of eggs in the liver leads to significant immune cell infiltration, and arachidonic acid, as an inflammatory mediator, might play a crucial role in driving the inflammatory response (Sonnweber et al., 2018). Additionally, research has suggested that arachidonic acid could activate neutrophil sphingomyelinase bound to the surface of Schistosoma, which then hydrolyzes sphingomyelin molecules in the lipid bilayer, potentially offering an effective treatment strategy for schistosomiasis (El Ridi et al., 2010; El Ridi and Tallima, 2013; Tallima et al., 2020). However, current research primarily focuses on *Schistosoma* larvae and adults, and whether arachidonic acid is effective against eggs still requires further investigation.

Phospholipids have multiple functions, including maintaining the normal structure and function of biological membranes, participating in cell signaling, and facilitating the absorption and transport of fats and fat-soluble fibers (Ecker and Liebisch, 2014). In normal liver tissue, PE (36:4) levels were relatively high, whereas PC (32:0) was more prevalent in granuloma regions. Alterations in lipid composition might influence the functionality of membrane proteins, including receptors, ion channels, and transporters. Research has shown that schistosomiasis affected the glucose metabolism of host, leading to the upregulation of glycolysis-related genes in the liver, such as *Ldha, Glut4, Pkm2, Glut1, Pfkfb3, Aldoc, HK2*, and *Pfk* and could also improve insulin resistance (Xu et al., 2019; Cai et al., 2021). Our results indicated that in liver regions outside the granulomas, levels of D-glucose and pyruvic acid were higher compared to those in normal mice, suggesting increased glucose metabolism. This might be due to a compensatory enhancement of glucose metabolism in the remaining liver cells, associated with granuloma formation and liver cell damage. Meanwhile, our results showed that taurine was increased in liver of schistosomiasis (Areas B and C). Taurine is a versatile amino acid that aids in bile production and secretion, while its antioxidant properties help neutralize free radicals and reduce oxidative stress damage to cells (McGaunn and Baur, 2023). Taurine has also been shown to improve liver granulomas and fibrosis in mice with schistosomiasis (Yu et al., 2016). Additionally, elevated Taurine might serve as a potential diagnostic marker for schistosomiasis (Wang et al., 2004). L-glutamine levels were reduced in the liver of schistosomiasis. Glutamine is one of the most abundant and versatile amino acids in the body, essential for maintaining cellular energy metabolism, nucleotide and amino acid biosynthesis, and redox balance (Vander Heiden et al., 2009). Additionally, it is crucial for lymphocyte proliferation, cytokine production, macrophage activation, and neutrophil bactericidal activity (Cruzat et al., 2018). During the formation of egg granulomas and liver fibrosis, the active inflammatory response of immune cells might lead to increased consumption of glutamine. Meanwhile, high levels of spermidine were observed in the liver of schistosomiasis. Spermidine plays a crucial role in regulating immune cells (Chamoto et al., 2024). It promotes a shift in macrophages towards an anti-inflammatory phenotype, which helps to reduce inflammation (Yang et al., 2016). Additionally, spermidine is essential for the differentiation of T cells, B cells, and natural killer cells, and it helps maintain their functional stability (Puleston et al., 2014; Zhang et al., 2019; O’Brien et al., 2021). Elevated spermidine levels might play a significant role in sustaining the function of immune cells in egg granulomas. Our results revealed elevated uric acid levels in the egg granulomas. Hypoxanthine is metabolized by xanthine dehydrogenase into xanthine, which is then further converted into uric acid. As the final product of purine metabolism, uric acid can accumulate excessively, leading to inflammatory arthritis and gout. However, research suggests that uric acid might also promote Th2 cell immunity by activating dendritic cells through spleen tyrosine kinase and PI3-kinase δ signaling pathway (Kool et al., 2011). Schistosomiasis is closely associated with type 2 immunity, with Th2 cells facilitating granuloma formation and liver fibrosis (Fairfax et al., 2012). Uric acid exhibits potential in promoting type 2 immunity, though its mechanisms require further investigation.

## Conclusions

Our study primarily explored the metabolic reprogramming of the liver in schistosomiasis through spatial localization. The differential metabolites across different liver regions were associated with pathways including the biosynthesis of unsaturated fatty acids, taurine and hypotaurine metabolism, glycerophospholipid metabolism, glycolysis/gluconeogenesis, purine metabolism, primary bile acid biosynthesis, arachidonic acid metabolism, and bile secretion. We observed many differential metabolites, such as unsaturated fatty acids, taurine, glutamine, spermidine, and uric acid, and their spatial distribution in the liver of schistosomiasis. We also hope that the spatial distribution of differential metabolites will provide valuable insights for understanding the pathogenesis and progression of schistosomiasis.

## Data Availability

All of data and materials are available.
